# Frailty, Nutrition, and Quality of Life in Urban‐Dwelling Older Adults Facing Vulnerability: Observational Study in Primary Heath Care Settings in Underpopulated Areas

**DOI:** 10.1111/nhs.70234

**Published:** 2025-09-20

**Authors:** Kalyne Patrícia de Macêdo Rocha, Larissa Amorim Almeida, Nathaly da Luz Andrade, Mayara Priscilla Dantas Araújo, Andreia Luíza de Oliveira, Maria Eduarda Oliveira de Albuquerque, Matheus Medeiros de Oliveira, Estefane Beatriz Leite de Morais, Rafaela Carolini de Oliveira Távora, Carola Rosas, Maria Antónia Fernandes Caeiro Chora, Maria Laurência Grou Parreirinha Gemito, Bruno Araújo da Silva Dantas, Gilson de Vasconcelos Torres

**Affiliations:** ^1^ Programa de Pós‐Graduação em Ciências da Saúde Universidade Federal do Rio Grande do Norte Natal Rio Grande do Norte Brazil; ^2^ Faculdade de Ciências da Saúde do Trairi Universidade Federal do Rio Grande do Norte Natal Rio Grande do Norte Brazil; ^3^ Departamento de Enfermagem Universidade Federal do Rio Grande do Norte Natal Rio Grande do Norte Brazil; ^4^ Universidad Austral de Chile Valdivia Chile; ^5^ Escola Superior de Enfermagem São João de Deus Universidade de Évora Évora Portugal

**Keywords:** elderly, frailty, nutritional impairment, primary health care, quality of life, vulnerability

## Abstract

To investigate the predictive relationships between frailty, nutritional factors, and Quality of Life (QoL) on the vulnerability of older adults enrolled in Primary Health Care (PHC) in an urban area. This was a cross‐sectional study conducted with community‐dwelling older adults. Participants receiving care in PHC in two Brazilian municipalities located in a sparsely populated region were recruited. The instruments used were Vulnerable Elders Survey (VES‐13), Mini Nutritional Assessment (MNA), Edmonton Frailty Scale (EFS), and Medical Outcomes Study Short Form‐36 (SF‐36). Association analyses, Spearman's correlation, and binary logistic regression were used. A total of 323 individuals were included. Binary logistic regression revealed frailty (EFS) as the main predictor of vulnerability (*R* ^2^ = 0.20; *p* < 0.001; OR = 1.35 [95% CI: 1.24–1.48]), with functional independence (*R* ^2^ = 0.25; *p* < 0.001; OR = 3.9 [95% CI: 2.74–5.73]) and functional performance (*R* ^2^ = 0.17; *p* < 0.001; OR = 3.21 [95% CI: 2.21–4.67]) being the domains that most strongly increased the odds of vulnerability. Impaired nutrition showed a consistent predictive association (*R* ^2^ = 0.11; *p* < 0.001; OR = 0.82 [95% CI: 0.76–0.89]). Frailty and poor nutritional status were predictors of vulnerability, with particular emphasis on physical‐functional aspects. QoL showed a moderate to strong correlation with vulnerability, especially in the physical domains.

**Summary**

- There was a correlation between older adults' vulnerability and impairments in nutritional status, quality of life, and the presence of frailty.
- Frailty was the main predictor of increased vulnerability among participants.
- Impairments in frailty domains related to functionality were associated with more than three times the odds of being classified as vulnerable.

## 1 Introduction

Frailty, a condition defined by functional, social, and cognitive decline, is also a key determinant of vulnerability, as it compromises autonomy and Quality of Life (QoL) (Navarrete‐Villanueva et al. 2021). It is estimated that between 10% and 25% of individuals over 65 years of age are frail, increasing to 45% among those aged 85 and older (Sousa et al. 2021). The literature shows that frail older adults are more likely to experience adverse health events such as falls, hospitalizations, and clinical deterioration compared to the general elderly population (Almeida et al. 2025; Zolnowski‐Kolp et al. 2023). Understanding this dynamic, particularly in conjunction with nutritional status and QoL, is essential to implementing preventive strategies and targeted interventions that may delay its effects among older adults (Tan et al. 2022).

One of the main factors that exacerbates vulnerability in this population is malnutrition, resulting from insufficient nutrient intake and associated with higher rates of morbidity and mortality (Urgessa 2022). Its prevalence ranges from 21% in community settings to 50% among hospitalized older adults (Brunner et al. 2022). Thus, nutritional aspects play a crucial role in maintaining physiological function, especially considering the natural decline in nutrient absorption during the aging process (Norman et al. 2021). Adequate dietary patterns are also associated with improved QoL, positive self‐perception of health, better clinical outcomes, and reduced mortality, factors that directly impact vulnerability (Chin et al. 2023).

QoL refers to the individual's subjective perception of their own existence, considering aspects such as physical and mental health, degree of autonomy, social interactions, and personal values (Ciconelli et al. 1999). A Brazilian study identified a significant association between functional aspects of QoL and vulnerability (Perseguino et al. 2021). Additionally, satisfaction with living conditions and access to health services are also factors that positively influence perceived QoL (Geigl et al. 2023).

Vulnerability is defined as the risk of functional decline or death within 2 years among older adults. This risk increases with advancing age, poorer self‐rated health, and reduced ability to perform activities of daily living, typically associated with impaired physical condition (Luz et al. 2015). Studies conducted in different regions of Brazil have reported prevalence rates ranging from 34.6% to 40.8%, affecting primarily women, individuals without partners, and those with chronic diseases and conditions (Gonzalez et al. 2023; Sousa et al. 2021). Although international studies on vulnerability remain scarce, researchers in France found a prevalence of 54.0% (Belmin et al. 2020), and other authors found that loss of muscle strength and dependence on activities of daily living were key factors in defining vulnerability (Pachołek and Sobieszczańska 2021). A Brazilian study confirmed its importance as a limiting factor for life expectancy among community‐dwelling older adults (Assis et al. 2021). However, its relationship with other important aspects of aging, such as frailty, nutrition, and QoL, has not been thoroughly explored.

Considering this, Primary Health Care (PHC) plays a central role in the early identification and prevention of frailty, nutritional deficits, and vulnerability. With a comprehensive and continuous approach, PHC enables ongoing monitoring of older adults' health status, supporting timely interventions that can reduce the risk of cognitive, functional, nutritional, and other declines (Ceccon et al. 2021).

Within this context, vulnerability has been approached in a fragmented manner, with limited integration or comparison with other key domains of aging. Likewise, factors such as frailty, nutritional status, and QoL are often evaluated in isolation, particularly within the scope of PHC. In response to this fragmentation, the present study seeks to fill these gaps by examining how vulnerability among older adults is shaped by the combined influence of frailty, nutritional impairments, and QoL outcomes. A deeper understanding of these interrelationships may contribute to more effective and comprehensive health policies targeting the needs of older individuals in primary care settings.

The objective of this study is to investigate the predictive relationships between frailty, nutritional factors, and QoL on the vulnerability of older adults enrolled in Primary Health Care in an urban area. The study hypothesizes that vulnerability is variably influenced by increased frailty, nutritional deficits, and impaired QoL among community‐dwelling older adults.

## 2 Materials and Methods

### 2.1 Study Design and Setting

This was an observational, cross‐sectional study with quantitative analysis methods, conducted among older adults receiving care in PHC services in the municipalities of Santa Cruz and Macaíba, in the state of Rio Grande do Norte, Northeast Brazil. Santa Cruz has an estimated older adult population of 5433, whereas Macaíba has 10 093, and both municipalities are considered to have low population density. This research is part of a series of studies developed by the international network [Blinded Network name], of which the authors are active members.

### 2.2 Population and Sample

The study population comprised older adults attending PHC services in the municipalities. Using G*Power 3.1, a priori power calculation was performed to estimate the required sample size for logistic regression (Hsieh et al. 1998). A statistical power of 80% was set to detect an Odds Ratio (OR) of 1.4, selected conservatively due to limited existing evidence on predictors of the outcome. With a significance level of 0.05, the analysis indicated that a minimum of 305 participants would be needed. The assumptions included a two‐tailed test and an expected prevalence of 41%, as suggested by previous literature (Gonzalez et al. 2023; Sousa et al. 2021). A non‐probabilistic, intentional, and convenience sampling method was used. A total of 323 individuals completed the study.

To be included, participants had to be 60 years or older, the legal threshold in Brazil for classification as an older adult (Brasil 2006); and had to be registered with or receiving care from a PHC unit for at least 6 months prior to data collection. Exclusion criteria included severe cognitive impairment diagnosed by healthcare professionals that prevented participation in the interview, and individuals receiving nutrition through non‐oral routes (e.g., enteral or parenteral feeding). Participants were recruited with the support of primary health care professionals from the municipalities where the study was conducted, totaling 30 units covering the entire urban area. These professionals provided addresses and contact information of eligible individuals based on their records. This facilitated participant identification by the researchers, in addition to those who were invited while attending appointments at the health units.

### 2.3 Instruments and Variables

Data were collected using structured questionnaires based on the variables of interest. The Elderly Health Handbook, a tool implemented in Brazilian PHC to monitor sociodemographic and health aspects of older adults (Brasil 2017), was used to extract data on sex, age group (60–79 and ≥ 80 years), skin color (White or non‐White), literacy, polypharmacy, and self‐reported chronic conditions, including stroke, anemia, asthma, diabetes mellitus, among others. These variables were selected to assess their association with the outcome, as previous studies have indicated a potential impact on vulnerability (Assis et al. 2021; Gonzalez et al. 2023; Sousa et al. 2022). Sociodemographic and health variables that showed an association and had a pre‐existing theoretical relationship with the outcome were considered eligible for adjusted analysis to examine the presence of confounding factors.

Vulnerability was assessed using the Vulnerable Elders Survey (VES‐13), which consists of 13 items across four domains: age, self‐rated health, physical limitations, and functional disabilities. The total score ranges from 0 to 10, with individuals scoring ≥ 3 classified as vulnerable (Luz et al. 2015). Accordingly, participants were categorized into “Vulnerable” (≥ 3 points) and “Non‐vulnerable” (< 3 points) groups. It was treated as the dependent and primary outcome variable in its categorical form. Its total score was used as a continuous variable for the relevant analyses. Internal consistency of the instrument was *α* = 0.62 (substantial).

Multidimensional aspects were represented by frailty, nutritional status, and QoL. Frailty was assessed using the Edmonton Frailty Scale (EFS), covering nine domains: cognition, general health, functional independence, social support, medication use, nutrition, mood, continence, and functional performance. Scores are originally classified as: Not frail (0–4), Apparent vulnerability (5–6), Mild frailty (7–8), Moderate frailty (9–10), and Severe frailty (> 11) (Fabrício‐Wehbe et al. 2009). For this analysis, the total score was used as a continuous variable, and internal consistency was *α* = 0.71 (substantial).

Nutritional assessment was performed using the Mini Nutritional Assessment (MNA), composed of 18 questions covering anthropometric, dietary, clinical, and self‐perceived health and nutrition domains. Total scores classify individuals as malnourished (< 17 points), at risk of malnutrition (17–23.5), or well‐nourished (24–30) (Acuña and Cruz 2004; Rubenstein et al. 2001). For this analysis, the total score was used as a continuous variable, and reliability was *α* = 0.75 (substantial).

QoL was measured using the Medical Outcomes Study Questionnaire Short Form 36 (SF‐36), which includes 36 items across eight domains: functional capacity, physical aspects, pain, general health, vitality, social aspects, emotional aspects, and mental health, grouped into two dimensions: physical health and mental health, scored on a scale from 0 to 100 (Ciconelli et al. 1999). Its score was used as a scalar variable for the relevant analyses. Reliability was *α* = 0.84 (almost perfect).

All instruments were adapted and validated for Brazilian Portuguese.

### 2.4 Data Collection

Data were collected face‐to‐face in Brazilian Portuguese using printed questionnaires between June 2023 and March 2024. Interviewers were undergraduate and graduate health sciences students from the participating university. All were trained by the project coordinators, who also supervised data collection. Interviews lasted approximately 60 min and were conducted at PHC units, participants' homes, or university facilities, depending on prior scheduling. Neither researchers nor participants were blinded.

### 2.5 Data Analysis

Data were analyzed using Statistical Package for the Social Sciences (SPSS) version 23.0. Descriptive analyses were performed using absolute and relative frequencies for categorical variables. Kolmogorov–Smirnov tests indicated non‐normal distribution. Pearson's chi‐square test was used for associations between categorical variables, and ORs were calculated to identify possible confounders in sociodemographic and health variables (OR > 1.00, with a 95% confidence interval).

Spearman's correlation was used to assess strength between the dependent variable (VES‐13) and the independent variables. Correlation strength was classified as: *r* < 0.29 (weak); 0.30 ≤ *r* ≤ 0.49 (moderate); *r* ≥ 0.50 (strong).

Binary logistic regression was primarily used to assess and compare the main predictors of vulnerability by individually analyzing the independent scalar variables related to the domains and aspects of nutritional status, frailty, and QoL. A hierarchical (stepwise) model was also implemented to assess the prediction of the outcome using a multivariate approach, as well as to examine the interaction between the independent variables. This choice was also made to enable a specific and detailed analysis of the predictive value of each domain of the multidimensional aspects, given that some of them include several variables representing distinct elements. Prediction was determined based on the relevance of *R* ^2^, the direction of the β coefficient, and the OR (95% CI), provided that *p* values were statistically significant. A multicollinearity analysis was performed to test the requirements of the variables, considering as eligible those with a tolerance value > 0.1 and a Variance Inflation Factor (VIF) < 10.0. Independent variables that showed higher OR and *R* ^2^ coefficients, as well as those representing the global scores of each scale used in the study, were included in an additional binary logistic regression model. This model was adjusted for sociodemographic and health variables considered eligible as potential influencers of vulnerability based on theoretical rationale and the results of the categorical association analyses. A variable was considered a confounding factor when the *R* ^2^ coefficient increased by more than 0.10 when comparing the crude and adjusted models (Cohen 2013).

Cronbach's alpha was used to assess internal consistency of instruments, with the following thresholds: *α* = 0–0.21 (slight); *α* = 0.21–0.40 (fair); *α* = 0.41–0.60 (moderate); *α* = 0.61–0.80 (substantial); *α* = 0.81–1.00 (almost perfect) (Landis and Koch 1977).

### 2.6 Ethical Considerations

This study was conducted in accordance with the Declaration of Helsinki on ethical research practices. It was reviewed and approved by the Research Ethics Committee of [Blinded Institution] (approval number 4.267.762), in accordance with national regulations for human subjects research. Authorization was granted by the local PHC authority prior to data collection. All participants provided written informed consent. For illiterate individuals, consent was given verbally, and fingerprint signatures were accepted. No compensation or incentives were offered for participation.

## 3 Results

The initial sample consisted of 323 individuals, of whom *n* = 148 (45.8%) were classified as vulnerable. The mean age was 73.1 (SD = 8.9) years, and no participants were excluded from the analysis.

Table 1 presents the comparative analysis of sociodemographic and health profiles between the study groups. The majority of the sample was female (*n* = 217; 67.2%), aged 60–79 years (*n* = 247; 76.5%), and single/widowed/divorced (*n* = 172; 53.3%). In the intergroup analysis, being aged 60–79 years and having the married/cohabiting marital status were identified as protective factors against vulnerability. Regarding health‐related variables, similar patterns were observed: The absence of polypharmacy and the presence of self‐reported chronic diseases were associated with the non‐vulnerable group. Based on these results and the existing theoretical framework, the variables age range and polypharmacy were considered for adjusted analyses to identify potential confounding factors.

**TABLE 1:** Sociodemographic and health characteristics of older adults in primary health care according to vulnerability status (*n* = 323).

Sociodemographic and health profile	Vulnerability (VES‐13)							
	Not vulnerable (*n* = 175)		Vulnerable (*n* = 148)		Total (*n* = 323)		*p* ^a^	OR (95% CI)^b^
	*n*	%	*n*	%	*n*	%		
Gender								
Woman	125	71.4	92	62.2	217	67.2	0.077	1.22 (0.97–1.54)
Man	50	28.6	56	37.8	106	32.8		
Age range, year								
60–79	151	86.3	96	64.9	247	76.5	< 0.001	1.93 (1.37–2.73)
≥ 80	24	13.7	52	35.1	76	23.5		
Marital status								
Single/widowed/divorced	84	48.0	88	59.5	172	53.3	0.040	1.23 (1.01–1.51)
Married/cohabitating	91	52.0	60	40.5	151	46.7		
Skin color								
White	72	41.1	59	39.9	131	40.6	0.816	1.02 (0.84–1.25)
No white	103	58.9	89	60.1	192	59.4		
Education								
Literate	141	80.6	108	73.0	249	77.1	0.105	1.23 (0.94–1.61)
Iliterate	34	19.4	40	27.0	74	22.9		
Fall history								
No	74	42.3	56	37.8	130	40.2	0.417	1.09 (0.89–1.33)
Yes	101	57.7	92	62.2	193	59.8		
Polypharmacy								
No	157	89.7	114	77.0	271	83.9	0.002	1.67 (1.14–2.46)
Yes	18	10.3	34	23.0	52	16.1		
Self‐reported chronic diseases								
No	44	25.1	15	10.1	59	18.3	0.001	1.50 (1.24–1.82)
Yes	131	74.9	133	89.9	264	81.7		

*Note:* Fall history refers to the self‐reported occurrence of accidental falls after the age of 60. Skin color non‐white includes individuals who self‐identified as Black, Brown, Indigenous, or Asian, grouped under a single variable due to their classification as presumably vulnerable ethnic groups. Polypharmacy: defined as the continuous daily use of five or more medications. Self‐reported chronic diseases include only conditions reported by participants, without requiring medical documentation or formal diagnosis confirmation.

Abbreviations: 95% CI, confidence interval 95%; EFS, Edmonton Frailty Scale; MNA, mini nutritional assessment; OR, odds ratio; QoL, quality of life; SF‐36, medical outcomes study questionnaire short form; VES‐13, vulnerable elders survey‐13.

^a^ Pearson's Chi‐Square test.

^b^ Odds ratio for cohort “Not vulnerable.”

Next, we analyzed the correlation between the scalar variables of vulnerability and the multidimensional aspects, presented in Table 2. In the analysis, nutrition (MNA) maintained a moderate correlation in both screening and global evaluations. Strong correlations were observed for the Functional Independence and Total Score domains of frailty (EFS), as well as for Physical Role Functioning and the Physical Health and Mental Health dimensions of QoL (SF‐36). However, most individual frailty domains showed weak correlations, whereas several QoL domains indicated moderate correlations with vulnerability (VES‐13).

**TABLE 2:** Correlation between vulnerability (VES‐13) and nutritional status, frailty, and QoL (*n* = 323).

Multidimensional aspects	Vulnerability (VES‐13)	
	*r* ^a^	*p* ^b^
Frailty (EFS)		
Knowledge	0.23	< 0.001
Previous hospitalization	0.10	0.065
Self‐perception of health	0.37	< 0.001
Functional independence	0.55	< 0.001
Social support	−0.12	0.033
Polypharmacy	0.25	< 0.001
Memory for medicines	0.21	< 0.001
Nutrition	0.12	0.024
Humor	0.15	0.005
Continence	0.14	0.011
Functional performance	0.45	< 0.001
Total score	0.50	< 0.001
Nutritional status (MNA)		
Screening	−0.32	< 0.001
Global assessment	−0.38	< 0.001
QoL (SF‐36) domains		
Physical role functioning	−0.55	< 0.001
Physical functioning	−0.40	< 0.001
Pain	−0.32	< 0.001
General health perceptions	−0.31	< 0.001
Vitality	−0.24	< 0.001
Social role functioning	−0.31	< 0.001
Emotional role functioning	−0.05	0.315
Mental health	−0.29	< 0.001
Total score	−0.49	< 0.001
Dimensions		
Physical health	−0.53	< 0.001
Mental health	−0.32	< 0.001

Abbreviations: EFS, Edmonton Frailty Scale; MNA, mini nutritional assessment; QoL, quality of life; SF‐36, medical outcomes study questionnaire short form; VES‐13, vulnerable elders survey‐13.

^a^ Correlation coefficient.

^b^ Spearman's correlation test. Correlation levels: *r* < 0.29 (weak); 0.29 ≥ *r* ≤ 0.49 (moderate); *r* ≥ 0.50 (strong).

Table 3 presents the results of the binary logistic regression analysis, which identified frailty (EFS) as the strongest predictor of vulnerability (VES‐13). Within frailty, the domains of Functional Independence and Functional Performance stood out, indicating that lower functional capacity in these domains increased the odds of being vulnerable by up to 3.96 times. The global nutritional assessment variable (MNA) also demonstrated predictive value, although with a lower and less consistent coefficient, indicating that higher scores are associated with reduced odds of vulnerability. None of the QoL variables were included in the model due to multicollinearity, as determined by the established parameters. For this reason, they were not eligible for analysis.

**TABLE 3:** Binary logistic regression between vulnerability and multidimensional aspects (*n* = 323).

Multidimensional aspects	Binary logistic regression—vulnerability (VES‐13)				
	*R* ^2^ ^a^	*p* ^b^	*ß* ^c^ (S.E)	Wald	OR (95% CI)
Frailty (EFS)					
Knowledge	0.04	0.003	0.37 (0.13)	8.62	1.45 (1.32–1.86)
Previous hospitalization	0.02	0.031	0.64 (0.29)	4.65	1.89 (1.06–3.37)
Self‐perception of health	0.10	< 0.001	0.77 (0.16)	23.24	2.16 (1.58–2.96)
Functional independence	0.25	< 0.001	1.38 (0.19)	53.49	3.96 (2.74–5.73)
Social support	0.03	0.008	−0.77 (0.29)	7.09	0.46 (0.26–0.81)
Polypharmacy	0.07	< 0.001	0.69 (0.17)	16.67	2.00 (1.43–2.78)
Memory for medicines	0.05	0.001	0.74 (0.22)	11.27	2.10 (1.36–3.24)
Nutrition	—^d^	—	—	—	—
Humor	0.03	0.009	0.60 (0.23)	6.79	1.82 (1.16–2.84)
Continence	—	—	—	—	—
Functional performance	0.17	< 0.001	1.17 (0.19)	37.18	3.21 (2.21–4.67)
Total score	0.20	< 0.001	0.30 (0.05)	43.39	1.35 (1.24–1.48)
Nutritional status (MNA)					
Screening	0.08	< 0.001	−0.25 (0.06)	19.45	0.78 (0.69–0.87)
Global assessment	0.11	< 0.001	0.19 (0.04)	24.26	0.82 (0.76–0.89)

*Note:* In the multicollinearity analysis, the variables from the MNA and EFS met the established criteria of tolerance (> 0.1) and VIF (< 10.0). However, all variables from the SF‐36 showed multicollinearity with the vulnerability outcome and were therefore not included in the model.

Abbreviations: 95% CI, confidence interval 95%; EFS, Edmonton Frailty Scale; MNA, mini nutritional assessment; OR, odds ratio; VES‐13, vulnerable elders survey‐13.

^a^ *R* ^2^ de Nagelkerke.

^b^ Model (forward LR).

^c^ Unstandardized coefficient.

^d^ The variable was not eligible for the regression model and, therefore, was not calculated.

We also performed a binary logistic regression analysis adjusted for potential confounding factors identified through associations presented in Table 1 and supported by prior theoretical evidence. The independent variables were selected based on their relevance as observed in Table 3. In addition, interactions between independent variables were examined in relation to the outcome, with respective adjustments. As shown in Table 4, all *p* values remained statistically significant. The interaction between the variables Functional Independence and Functional Performance (EFS) was the most relevant, although it did not differ significantly from the parameters observed when these variables were analyzed individually. Nevertheless, the correlation coefficient (*R* ^2^) increased, showing a difference greater than 0.10 in most models adjusted for polypharmacy (Yes), suggesting improved explanatory power for the outcome without a substantial or consistent increase in the odds ratio after adjustment.

**TABLE 4:** Binary logistic regression was adjusted for potential confounding factors and interactions between independent variables in relation to the vulnerability outcome (*n* = 323).

Multidimensional aspects	Binary logistic regression—vulnerability (VES‐13)				
	*R* ^2^ ^a^	*p* ^b^	*ß* ^c^(S.E)	Wald	OR (95% CI)
Frailty (EFS)					
Total score—crude	0.20	< 0.001	0.30 (0.05)	43.39	1.35 (1.24–1.48)
Adjusted by					
Age range 60–79 years	0.16	< 0.001	0.27 (0.05)	26.90	1.30 (1.18–1.44)
Age range ≥ 80 years	0.25	0.001	0.38 (0.11)	11.02	1.46 (1.67–1.83)
Polipharmacy (no)	0.14	< 0.001	0.26 (0.05)	25.97	1.29 (1.17–1.43)
Polipharmacy (yes)	0.42	0.001	0.60 (0.18)	10.73	1.82 (1.27–2.61)
Interactions					
Functional independence (EFS) with functional performance (EFS)	0.31	< 0.001	1.35 (0.22)	38.45	3.87 (2.52–5.93)
Adjusted by					
Age range 60–79 years	0.24	< 0.001	1.26 (0.25)	24.98	3.53 (2.15–5.79)
Age range ≥ 80 years	0.34	< 0.001	1.30 (0.43)	9.29	3.68 (1.59–8.50)
Polipharmacy (no)	0.25	< 0.001	1.34 (0.24)	29.93	3.81 (2.36–6.15)
Polipharmacy (yes)	0.44	< 0.001	1.32 (0.49)	7.12	3.74 (1.42–9.87)
Nutritional status (MNA)					
Global assessment—Crude	0.11	< 0.001	0.19 (0.04)	24.26	0.82 (0.76–0.89)
Adjusted by					
Age range 60–79 years	0.08	< 0.001	−0.16 (0.04)	14.47	0.85 (0.78–0.92)
Age range ≥ 80 years	0.24	0.001	−0.40 (0.13)	9.96	0.67 (0.52–0.86)
Polipharmacy (no)	0.08	< 0.001	−0.18 (0.04)	15.82	0.84 (0.77–0.91)
Polipharmacy (yes)	—^d^	—	—	—	—
Interactions					
Total score (EFS) with MNA‐global assessment	0.19	< 0.001	0.01 (0.00)	40.08	1.01 (1.01–1.02)
Adjusted by					
Age range 60–79 yeras	0.15	< 0.001	0.01 (0.00)	24.97	1.01 (1.01–1.02)
Age range ≥ 80 yeras	0.19	0.003	0.01 (0.00)	8.99	1.01 (1.01–1.02)
Polipharmacy (no)	0.13	< 0.001	0.01 (0.00)	24.45	1.01 (1.01–1.02)
Polipharmacy (yes)	0.39	0.001	0.03 (0.01)	10.55	1.03 (1.01–1.05)

*Note:* An interaction analysis involving all variables simultaneously (Functional independence, Functional performance, Total EFS score, and MNA—Global assessment) was conducted, but due to model complexity, SPSS did not consider them eligible and was unable to perform the calculation.

Abbreviations: 95% CI, confidence interval 95%; EFS, Edmonton Frailty Scale; MNA, mini nutritional assessment; OR, odds ratio; VES‐13, vulnerable elders survey‐13.

^a^ *R* ^2^ de Nagelkerke.

^b^ Model (forward LR).

^c^ Unstandardized coefficient.

^d^ The variable was not eligible for the regression model and, therefore, was not calculated.

Additionally, we attempted to implement a hierarchical binary logistic regression model (Stepwise) using the variables presented in Table 4. However, as the model increased in complexity, the statistical software did not consider it eligible and was unable to perform the analysis, producing no results beyond those already presented in the previous tables.

## 4 Discussion

The main findings of this study showed that frailty and poor nutritional status were the primary predictors of vulnerability (VES‐13). More refined analyses revealed that impairments in functional aspects of frailty increased the odds of vulnerability by more than threefold. Although physical and functional aspects of QoL were strongly correlated with vulnerability, they did not demonstrate consistency in more robust analyses. These results may support the development and implementation of more effective public health interventions and preventive care strategies, as well as guide the creation of new protocols for geriatric assessment.

When analyzing sociodemographic and health variables, we found that younger age, having a partner, and the absence of polypharmacy and chronic diseases acted as protective factors against vulnerability when comparing study groups. These findings are consistent with previous studies conducted in Brazil (Assis et al. 2021; Gonzalez et al. 2023; Sousa et al. 2022). Although our binary logistic regression analyses remained statistically significant after adjusting for age, we did not observe substantial increases in the predictive values for outcomes.

In terms of multidimensional aspects, when evaluating frailty, the correlation analysis revealed the highest coefficients for the domains of functional independence and functional performance, which were also the strongest predictors in the logistic regression, increasing the odds of vulnerability by more than threefold. This functional impairment related to frailty is well‐documented in the literature, primarily due to its physiological impact on the individual (Doody et al. 2023; Kim and Rockwood 2024). Accordingly, previous studies have indicated that frailty also increases the likelihood of traumatic events such as falls, which carry a high risk of morbidity and mortality (Qin et al. 2021). For these reasons, expert consensus guidelines recommend muscle‐strengthening exercises to improve functional capacity and reduce frailty (Izquierdo et al. 2021), with successful outcomes reported in other settings (Lin et al. 2020). These findings suggest that improving such aspects may also have a positive impact on the individuals included in our study.

When analyzing nutritional status, we found a moderate correlation with vulnerability. Binary logistic regression indicated that its predictive value was statistically significant, although it had a low explanatory power for the outcome. In the analysis of its interaction with frailty, a slight increase in its coefficient was observed, without suggesting relevant influences. These findings are consistent with those of another Brazilian study, which showed that malnourished participants also exhibited functional impairments (Cabral et al. 2018).

Regarding nutrition, previous authors have explained that age‐related physiological changes, such as reduced nutrient absorption, make older adults more susceptible to malnutrition (Norman et al. 2021). This condition contributes to complications including infections, falls, pressure injuries, institutionalization, and mortality, all of which negatively affect QoL in this population (Roberts et al. 2021). These findings highlight the importance of nutritional assessment in primary health care settings, particularly when considering the risk of vulnerability (Katsas et al. 2020).

Although literature on this specific association remains limited, the theoretical framework adopted in our study links vulnerability to self‐rated health and to physical limitations in performing activities requiring minimum physical conditioning (Luz et al. 2015), a trait also commonly found among well‐nourished older adults (Chen et al. 2023). A study conducted in Singapore showed that malnourished older adults had reduced physical conditioning and greater difficulty in performing daily activities (Ge et al. 2020), suggesting a possible indirect relationship between nutrition and vulnerability. However, in our logistic regression analysis, although nutritional impairment emerged as a predictor of vulnerability, it did not exhibit a high explanatory value for this outcome.

A Spanish intervention study highlighted that improvements in nutritional status also contributed to the reduction of frailty through educational interventions conducted via telephone (Casals et al. 2023). This finding supports the notion that frailty may also be influenced by nutritional factors, which may help explain the result observed in our interaction analysis between these variables.

In this context, our findings also demonstrated a strong correlation between vulnerability and aspects of QoL, although these variables were not eligible for binary logistic regression analysis. Beyond the previously discussed importance of these factors, it is worth noting that functional incapacity is associated with a progressive loss of physical and mental abilities, often linked to multimorbidity, depression, and difficulty managing daily activities. These limitations undermine the well‐being and autonomy of older adults and emphasize the need for public health measures aimed at supporting physical and social functioning in this population (Malik 2022), as also observed in a study conducted in Brazil and Portugal (de Miranda et al. 2022). Previous multidimensional interventions have shown positive outcomes in these QoL‐related aspects within primary health care settings, using simple and accessible strategies (Dantas et al. 2020), which may serve as feasible options for professionals working in contexts similar to ours.

Internationally, vulnerability remains an underexplored topic in scientific literature. Its dimensions could be more thoroughly addressed in global health policies as a critical component of older adult health. Based on our findings, we suggest that nutrition, specific aspects of frailty, and QoL, particularly physical and functional domains, may influence vulnerability in a broader sense. However, the external validity of our findings should be considered with caution, as this was a cross‐sectional study conducted in a geographically and culturally specific region of Brazil, limiting the applicability of these results to similar populations only.

## 5 Conclusion

This study found that frailty and poor nutritional status were predictors of vulnerability, with physical‐functional aspects standing out in the logistic regression model. QoL showed a moderate to strong correlation with vulnerability, particularly in physical domains, although it was not eligible for more refined analyses. The findings are consistent with the hypothesis originally proposed in this study.

### 5.1 Implications for Policy and Practice

Based on our evidence, PHC professionals and health managers across various disciplines may consider implementing screening and prevention strategies for vulnerability in older adults. These strategies should incorporate assessments of nutritional status, frailty, and QoL, particularly focusing on maintaining or improving physical and functional health. Although the generalizability of our data is limited, healthcare professionals working in similar settings can use the same instruments we implemented to assess these aspects in their own populations.

### 5.2 Limitations

This study presents some important limitations. The non‐probabilistic convenience sampling method may have limited sample representativeness, making it difficult to generalize the findings to other older adult populations in PHC. The cross‐sectional design does not allow for the establishment of causal relationships, restricting the analysis to associations only. In addition, relying on self‐reported data may introduce response bias, as it requires adequate comprehension of the questions, which may not occur consistently, especially among illiterate individuals included in our sample. Participants may also feel compelled to withhold information that they perceive as embarrassing, such as acknowledging various limitations.

Despite these limitations, significant methodological efforts were made to minimize potential biases. Researchers underwent thorough training to adapt the phrasing of questions using culturally familiar expressions, thereby facilitating comprehension, including for participants who were illiterate. The interviews were conducted in private and quiet settings to ensure the participants' comfort and confidentiality. They were always carried out with the presence of only the interviewer and the participant, avoiding environments where others could overhear the conversation. The search for a representative sample of the target population helped reduce selection bias, as we exceeded the minimum sample size estimated during the study design. Moreover, the statistical analyses were adjusted for confounding variables, strengthening the robustness of the findings by controlling for external interference in the associations observed.

## Author Contributions

**Kalyne Patrícia de Macêdo Rocha:** conceptualization, investigation, methodology, writing – original draft preparation. **Larissa Amorim Almeida:** conceptualization, data curation, investigation. **Nathaly da Luz Andrade:** conceptualization, data curation, investigation. **Mayara Priscilla Dantas Araújo:** conceptualization, data curation, investigation. **Andreia Luíza de Oliveira:** conceptualization, investigation. **Maria Eduarda Oliveira de Albuquerque:** conceptualization, investigation. **Matheus Medeiros de Oliveira:** conceptualization, investigation. **Estefane Beatriz Leite de Morais:** conceptualization, investigation. **Rafaela Carolini de Oliveira Távora:** methodology, supervision, validation. **Carola Rosas:** methodology, supervision, validation. **Maria Antónia Fernandes Caeiro Chora:** methodology, supervision, validation. **Maria Laurência Grou Parreirinha Gemito:** methodology, supervision, validation. **Bruno Araújo da Silva Dantas:** methodology, resources, supervision, visualization, writing – review and editing. **Gilson de Vasconcelos Torres:** data curation, formal analysis, funding acquisition, project Administration, validation, writing – review and editing.

## Ethics Statement

The study followed the principles of the Declaration of Helsinki and was approved by the Research Ethics Committee of the Onofre Lopes University Hospital, Federal University of Rio Grande do Norte, under approval number 4.267.762, in accordance with national regulations for research involving human subjects. The institution responsible for Primary Health Care (PHC) in the municipality granted prior authorization for the study.

## Consent

All participants read and signed an informed consent form. For illiterate individuals, the researchers read the consent form aloud and explained the main objectives and data collection procedures. Fingerprint impressions were accepted in place of written signatures when participants were unable to sign. No incentives or rewards were offered for participation in the study.

## Conflicts of Interest

The authors declare no conflicts of interest.

## Data Availability

The data that support the findings of this study are openly available in Mendeley Data, available from: https://data.mendeley.com/datasets/2yh5fp9yfk/1.
